# Metastasizing pleomorphic adenoma of the parotid gland

**DOI:** 10.3332/ecancer.2017.758

**Published:** 2017-08-15

**Authors:** Javier Soteldo, Nathasha Aranaga

**Affiliations:** Hospital de Clinicas, Piso 2, Consultorio 228, San Bernardino, Caracas, 1010 584248518639/2125086228, Venezuela

**Keywords:** metastasizing pleomorphic adenoma, salivary glands, parotid gland, adenoma, case report, bibliographic review

## Abstract

Salivary gland tumours are estimated to represent approximately 3% of all head and neck tumours. About 70–80% of these neoplasms occur in the major salivary glands, with the parotid gland being the most commonly affected site. The metastasizing pleomorphic adenoma (MPA) has histological characteristics of pleomorphic adenoma, but it has the capacity to generate local recurrences and distant metastases (mainly bones, lungs, and lymph nodes). Despite the fact that some authors consider it to be a benign neoplasia, the 2015 World Health Organisation (WHO) classification of head and neck tumours considers it to be malignant.

We present a highly unusual case of metastasizing pleomorphic adenoma of the parotid gland and a bibliographic review.

## Introduction

The metastasizing pleomorphic adenoma, also known as a metastasizing benign tumour and mixed malignant tumour, is a salivary gland tumour that histologically corresponds to a pleomorphic adenoma, but with the possibility of local lymph nodes and distant metastasis. The time of appearance of metastasis is between 1.5 and 55 years after surgery [1].

Most metastases from MPA occur in patients who underwent surgery one or more times, and a hypothesis is that during the surgery the cells tumour could spread to distant sites through the circulation, including bone (50%), lymph nodes (30%), lung (30%) and rarely spread to liver, central nervous system and skin [2, 3].

The most frequent location is in the parotid gland (79% of the cases), followed by the submaxillary gland (13%), and the palate (9%). It has been observed in females and males in their third and sixth decade of life [1, 4].

Clinically, it appears as a slow growing, painless volume enlargement in the parotid. A rapid growth and compression symptoms of the facial nerve are associated with malignancy [4].

These lesions are rounded, hypoechoic and well-defined lobed contours on ultrasound. On computerised tomography (CT), we can observe it as homogeneous, smooth and well defined, with greater higher attenuation than the surrounding parotid parenchyma. The size is variable; however, the small lesions are more circumscribed and homogeneous, while the large lesions can have lobulated borders, with enhancement, calcifications, and necrotic areas and/or haemorrhagic areas [5, 6].

Histologically, the primary salivary gland tumour and metastases are composed of the typical mixture of benign appearing epithelial and mesenchymal components of a pleomorphic adenoma. The histology is not predictive regarding its ability to metastasize. Mitotic figures and nuclear pleomorphism may be seen, but the tumour is not overtly histologically malignant [1].

In cases of recurrent or long-standing pleomorphic adenomas, a high index of suspicion for MPA is advisable, complementary studies should be considered to rule out metastases by full body CT, magnetic resonance, and/or PET [4].

The most widely accepted procedure is total parotidectomy with conservation of the facial nerve unless this has been infiltrated by the tumour. The decision on the most appropriate treatment is taken by intraoperative confirmation. The application of radiotherapy is considered as complementary treatment [7].

The prognosis of the MPA justifies its classification as a malignant neoplasia, because its mortality at 5 years reaches 50% [4].

## Case report

A 36-year-old Caucasian male presented to our practice with a palpable node on the back of the neck and a painless progressive augmentation volume on the left cervical region, with no other concomitant symptom, that started 5 years earlier. The patient underwent excision of pleomorphic adenoma of the parotid gland 18 years earlier.

The physical examination evidenced a painless volume enlargement on the left parotid region and a 1 cm nodule on the back of the neck.

**Figure 1.**
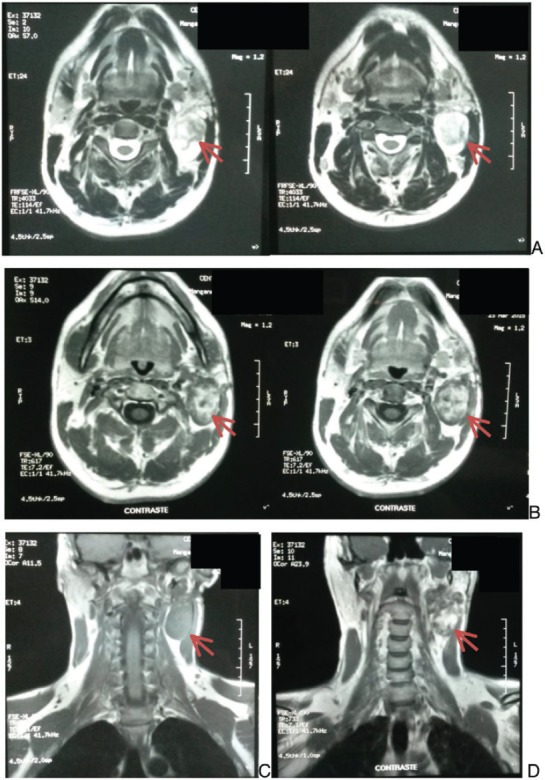
Head and neck magnetic resonance. A. Cross section without contrast. B. Cross section with contrast. C. Coronal section without contrast. D. Coronal section with contrast. Arrow indicates lesion.

A head- and neck-contrasted magnetic resonance shows: a lesion that completely occupies the left parotid gland, 5.8 × 4 × 3 cm, presenting adjacent pathological nodular activity, of approximately 5 mm that occupies the posterior fatty space of the sternocleidomastoid muscle (Figure 1).

**Figure 2.**
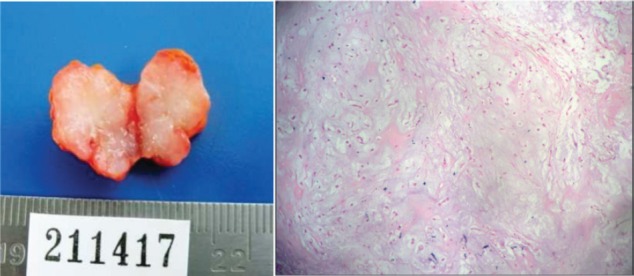
Excisional biopsy. Cervical adenopathy level II. Histological findings.

After submitting informed consent, an excisional biopsy of the left cervical adenopathy is taken (level II) that concludes: pleomorphic adenoma. Components of lymph nodes are not observed, and thus, a recurrent pre-existing lesion is suggested or a variation of the metastasizing pleomorphic adenoma (Figure 2).

A total left parotidectomy was performed, also a selective left side cervical dissection of levels IA and II, with the informed consent of the patient.

**Figure 3.**
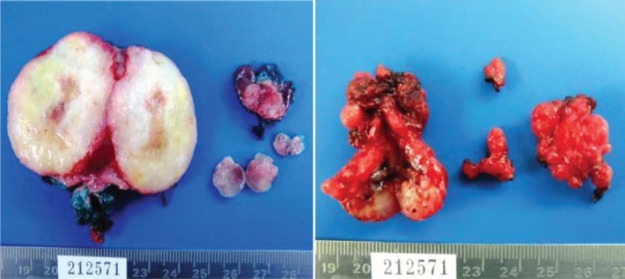
Surgical specimen. Total left parotidectomy. Nodular levels IA and II.

**Figure 4.**
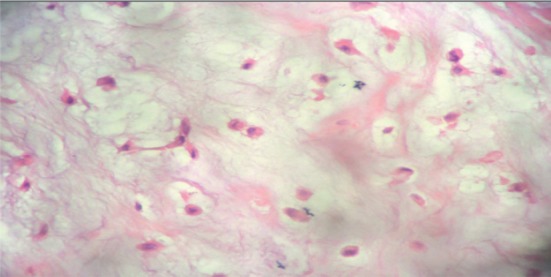
Surgical specimen. Total left parotidectomy. Histological findings.

The result of the surgical specimen reports multinodular myxoid recurrent pleomorphic adenoma (Figure 3 and 4).

Two years have passed since the surgical intervention; the patient is currently disease free, he performs regular monitoring where physical examination and ultrasound are performed.

There were no adverse or unanticipated events to report.

## Discussion

The metastasizing pleomorphic adenoma is a salivary gland tumour that is observed with very low frequency and that has a malignant behaviour, potentially lethal while its histology continues to be benign [4].

In our bibliographic review, we were able to observe that despite some authors considering this neoplasia to be benign, the 2015 World Health Organisation (WHO) classification of head and neck tumours considers it to be a malignant tumour of epithelial origin. It is important to point out that the International Classification of Diseases for Oncology (ICD-O), assigns a behavioural code /1 that corresponds to neoplasia of uncertain and unknown behaviour [1, 9].

The statistics for this pathology are still unknown and some aspects continue to be controversial. McGarry et al., in his 2015 publication, analyses a period of 50 years, and observed until 2007 only 52 cases, while Knight et al performed a systemic revision of the bibliography and found that from 1942 to 2014, there were 80 known cases worldwide, showing that the average age for diagnosis was 49.5 years; the patient of our publication was 36 years old. The study of LiVolsi and Perzin reviewed 47 patients with an average age of 59 (34–86), with an incident of more women than men, 35 and 12 cases, respectively [4, 8, 10].

The most frequent localisation of the primary tumour is in the parotid gland, followed by the submaxillary gland, the palate, and the minor salivary glands.

As established in the literature, the most frequent clinical presentation is the painless augmentation of the volume in the affected salivary gland, found in 39 of the 47 cases of LiVolsi and in the 80 cases of Knight´s series, as well as in our patient [8, 10].

The latency period between the presentation of the initial pleomorphic adenoma and the detection of the recurrence or distant metastasis was of 16 years for McGarry and 14.9 for Knight. A case published by Bonet-Loscertales et al in 2010 presented double recurrence, the first at 7 years and the second at 14 years of the first diagnosis. In our case, it presented at 18 years after the enucleation of the first adenoma [4, 8, 11].

There are several hypotheses about the aetiology of the metastasis and the recurrence. It is believed that the enucleation of the pleomorphic adenoma may be incomplete contributing to recurrence, during surgery vascular implantation of tumour cells can occur, favouring the dissemination by haematogenous route. The metastases are observed more frequently in bones, lung, and lymph nodes. Young et al discovered a case of hepatic metastasis, McGarry in the supraspinatus muscle, in the revision of Knight et al the metastases were distributed as follows: bone 36.6%, lung 33.8 and cervical lymph nodes 20.1%, coinciding with our case report [2, 4, 8].

The treatment used in the majority of the cases for all the series was total parotidectomy conserving the facial nerve, if that structure was not infiltrated [1, 2, 7, 11].

Mortality at 5 years reaches 50%. The WHO reports that 40% of the patients die with the disease, 47% live free of disease, and 13% live with it [1, 4].

A recent study, undertaken by Mariano et al, published in September 2015, presents an interesting conclusion, it demonstrates the clonal origin of the MPA. Multiple genomic alterations are evidenced and could explain the metastatic nature of these lesions, which without a doubt promotes the realisation of future studies, because even though the frequency of this pathology is very low, its potential lethality should not be ignored [12].

## Conclusion

MPA is a low-grade malignant tumour, which histologically corresponds to a pleomorphic adenoma, but with the possibility of local lymph nodes and distant metastasis that shows a 50% mortality at 5 years. The WHO reports that 40% of the patients die with the disease, 47% live free of disease, and 13% live with it. There is a limited number of reported cases and some controversial aspects about MPA that make this entity a target for further studies.
